# Chronic Treatment with a Promnesiant GABA-A **α**5-Selective Inverse Agonist Increases Immediate Early Genes Expression during Memory Processing in Mice and Rectifies Their Expression Levels in a Down Syndrome Mouse Model

**DOI:** 10.1155/2011/153218

**Published:** 2011-10-19

**Authors:** J. Braudeau, L. Dauphinot, A. Duchon, A. Loistron, R. H. Dodd, Y. Hérault, B. Delatour, M. C. Potier

**Affiliations:** ^1^Centre de Recherche de l'Institut du Cerveau et de Moelle Epinière, INSERM UMRS 975, CNRS UMR7225, UPMC, 75013 Paris, France; ^2^Institut de Génétique et de Biologie Moléculaire et Cellulaire (IGBMC), Institut National de Santé et de Recherche Médicale (INSERM) U964/Centre National de Recherche Scientifique (CNRS) UMR 1704/Université de Strasbourg, 67404 Illkirch, France; ^3^Institut de Chimie des Substances Naturelles-CNRS UPR 2301, 91198 Gif-sur-Yvette, France; ^4^Groupe d'Intérêt Economique-Centre Européen de Recherche en Biologie et en Médecine (GIE-CERBM), Institut Clinique de la Souris (ICS), Université de Strasbourg, 67404 Illkirch, France

## Abstract

Decrease of GABAergic transmission has been proposed to improve memory functions. Indeed, inverse agonists selective for *α*5 GABA-A-benzodiazepine receptors (*α*5IA) have promnesiant activity. Interestingly, we have recently shown that *α*5IA can rescue cognitive deficits in Ts65Dn mice, a Down syndrome mouse model with altered GABAergic transmission. Here, we studied the impact of chronic treatment with *α*5IA on gene expression in the hippocampus of Ts65Dn and control euploid mice after being trained in the Morris water maze task. In euploid mice, chronic treatment with *α*5IA increased IEGs expression, particularly of *c-Fos* and * Arc* genes. In Ts65Dn mice, deficits of IEGs activation were completely rescued after treatment with *α*5IA. In addition, normalization of *Sod1* overexpression in Ts65Dn mice after *α*5IA treatment was observed. IEG expression regulation after *α*5IA treatment following behavioral stimulation could be a contributing factor for both the general promnesiant activity of *α*5IA and its rescuing effect in Ts65Dn mice alongside signaling cascades that are critical for memory consolidation and cognition.

## 1. Introduction

Down syndrome (DS) affects 0.45% of human conceptions [1] and is the first cause of mental retardation. This disorder is induced by total or partial trisomy of human chromosome 21 (HSA21) that delays both physical and mental development of affected children. In particular, cognitive skills, including learning and memory functions, are severely impaired in DS subjects.

Although being the focus of intense research activity [2, 3], attempts at developing treatments for counteracting cognitive defects in DS patients have not yet been successful. 

Since fifteen years, DS animal models have been engineered to mimic DS physiopathogeny. Ts65Dn mice [4], one of the most studied DS models, have 167 three-copy genes corresponding to more than half of the genes from HSA21. These mice develop gradual learning and memory impairments when compared to euploid animals (for review, see [5]) in conjunction with morphological anomalies. In addition, Ts65Dn mice show abnormal synaptic plasticity as exemplified by long-term potentiation (LTP) deficits [6]. 

Data from recent years strongly suggest that changes in LTP and associated learning and memory function in DS mice might result from an imbalance between excitatory and inhibitory neurotransmission. More precisely, it has been demonstrated that increased GABAergic activity in the brain of Ts65Dn mice could be responsible for altered cognitive phenotypes [7, 8], opening new avenues for therapeutic opportunities. Treatments relying on GABA-A antagonists such as picrotoxin and pentylenetetrazole (PTZ) have indeed rescued deficits in DS mice; GABA-A antagonists can restore normal LTP [8] and also normalize cognitive phenotypes in learning tests such as the novel object recognition [9] and Morris water maze [10]. Altogether, these studies suggest the potential use of GABA antagonists for stimulating cognitive performances in DS subjects. However, it is known that such drugs also have convulsant effects which definitively preclude their use as cognitive enhancers in humans.

As an alternative to GABA-A antagonists, GABA-A inverse agonists such as *β*-carbolines acting at the benzodiazepine recognition site decrease the efficacy of GABAergic transmission and have promnesiant effects [11–14]. Their use in humans is, however, hampered by their convulsant/proconvulsant and anxiogenic side effects [15, 16].

It is nonetheless assumed that various pharmacological profiles can be obtained using ligands with specific affinities for the different *α*1, *α*2, *α*3, or *α*5 benzodiazepine receptor subtypes [17] that are unevenly distributed in the brain [18–20]. It is known that the *α*5 subunit-containing receptors are largely expressed in the hippocampus [21, 22], a brain region involved in learning and memory processes that is dysfunctional in DS individuals [23, 24]. In mice, invalidation or mutation of the gene coding for the *α*5 subunit potentiate synaptic plasticity [25] and concurrently improve cognitive performances [25–27] without inducing anxiogenic or proconvulsant/convulsant side effects [28–32]. Recently, we demonstrated that acute and chronic treatment with an *α*5-selective inverse agonist, and referred to herein as compound *α*5IA, initially developed by Merck Sharp and Dohme Research Laboratories [33, 34] can restore cognitive deficits in a DS mouse model [35]. We further showed that following treatment with *α*5IA, the immediate early gene (IEG) product Fos is selectively increased in brain regions involved in learning and memory in control and DS mice [35].

In order to obtain insights into gene regulation pathways involved in the pharmacological effect of *α*5IA, we studied gene expression profiles in hippocampi of euploids and Ts65Dn mice behaviorally exposed to the Morris water maze (MWM) task, and treated or not with *α*5IA. We found that chronic treatment with *α*5IA globally increases the expression of IEGs and in particular of *c-Fos* and *Arc*. These effects could be related to the memory-enhancing properties classically described for *α*5IA [28–32]. 

In Down syndrome mice, we observed an abnormally low level of IEG induction after behavioral stimulation. In addition, some three-copy genes were significantly overexpressed, including *Sod1* gene. Chronic treatment of Ts65Dn mice with *α*5IA normalized the expression level of *Sod1* and in parallel restored a physiological level of IEGs expression. This double-action mode can explain the rescuing effect observed following *α*5IA treatment in DS mice. 

## 2. Material and Methods

### 2.1. Animals

**Figure 1:**
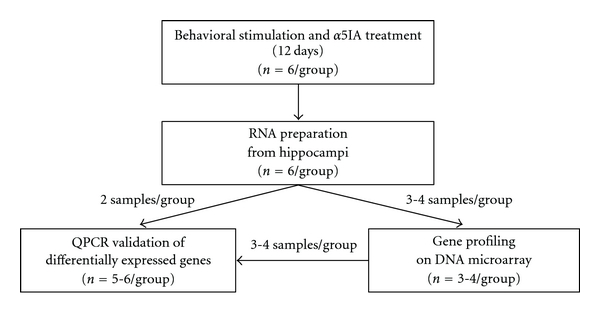
Experimental design used for the genomic studies: microarray and QPCR.

Male mice were produced at the Intragene resource center (TAAM, CNRS UPS44 Orléans, France) and bred on a mixed genetic background B6C3〈B〉, derived from C57BL/6J (B6) and a congenic inbred line C3H/HeH for the BALB/c wild-type Pde6b allele [36], thus avoiding retinal degeneration and impaired visual acuity. On this background, Ts65Dn mice showed similar behavioral phenotypes when compared to the original Ts65Dn line (AD and YH, personal communication; see also [37]). Mice were acclimated in our animal facility for at least 2 weeks before initiating behavioral testing. All experiments were conducted in accordance with the ethical standards of French and European regulations (European Communities Council Directive of 24 November 1986). A total number of 24 mice (12 Ts65Dn and 12 euploid mice) were behaviorally trained in the MWM task. Only a subset of these animals (7 and 10-11 mice per genotype for microarray and QPCR analysis, resp.) were processed for the molecular biology analysis described in the present work, (Figure 1).

### 2.2. *α*5IA Synthesis and Formulation

The drug used is 3-(5-methylisoxazol-3-yl)-6-(1-methyl-1,2,3-triazol-4-yl)methyloxy-1,2,4-triazolo[3,4-a]phthalazine (*α*5IA). It was synthesized by Orga-Link SARL (Magny-les-Hameaux, France), according to Sternfeld and collaborators [34]. The hydrochloride salt was prepared by dissolving the base in hot ethanol and adding a solution of 5% hydrochloric acid in ethanol until the solution was slightly acidic. Upon cooling, a precipitate formed which was collected by filtration, washed with cold ethanol, and dried.

The HCl salt of *α*5IA was solubilized in a mixture of DMSO, Cremophor El (BASF, Ludwigshafen, Germany), and hypotonic water (ProAmp) (10 : 15 : 75). *α*5IA or vehicle (solubilization solution) was injected intraperitoneally (i.p.) at 5 mg/kg [35].

### 2.3. Morris Water Maze

Cognitive stimulation was performed during 12 consecutive days in the MWM as previously described [35]. In brief, training consisted in a goal-location task in a pool (2–4 learning trials per daily sessions). Euploid and Ts65Dn mice were injected each day with either vehicle or *α*5IA (6 mice per group), 30 min before starting the session. Distance travelled to find the platform has been used as learning index. We compared the performances of vehicle-treated Ts65Dn mice to the 3 other groups (*α*5IA-treated Ts65Dn mice, vehicle-treated euploids, and *α*5IA-treated euploids) using Student's *t*-tests. Statistical significance was set to a *P* value <0.05.

### 2.4. cRNA Probe Preparation and Hybridization

Twenty five min following the last MWM training session, animals were sacrificed, and their brains were extracted and processed for gene expression profiling. Total RNAs were obtained from frozen individual hippocampi and treated with DNAse using NucleoSpin RNA II kit (Macherey Nagel, France) in accordance with the manufacturer's protocol. The quality and quantity of each RNA preparation were assessed on the Agilent 2100 Bioanalyzer with RNA 6000 NanoChips (Agilent Technologies).

Hundred ng of each RNA were amplified and labelled with Cy3 using the Low Imput Quick Amp labeling kit (Agilent Technologies) according to the manufacturer's instructions. After purification and quantification on a Nanoview (ThermoFisher Scientific), 2 *μ*g of each Cy3-cRNA were hybridized overnight on Whole Mouse Genome 4 × 44 K v1 Microarrays (Agilent Technologies) according to the manufacturer's instructions. 

### 2.5. Microarray Expression Data Analysis

Microarrays data were acquired on an Innoscan 900 (Innopsys, France) with a resolution of 2 *μ*m and analyzed with Mapix 5.0.0 software (Innopsys, France). For each array, raw data consisted of the median feature intensity and background feature intensity (F-B) at wavelength 532 nm. These raw data were log⁡2 transformed and quantile normalized under the R freeware (http://www.r-project.org/). Analysis of variance (ANOVA) with two main factors, Genotype (Ts65Dn versus euploid) and Treatment (*α*5IA versus vehicle), was then performed on the normalized data using the software MeV 4.6.2 (http://www.tm4.org/mev/). Gene ontology (GO) category enrichment analysis was realized using the web-based GOrilla application (http://cbl-gorilla.cs.technion.ac.il/). Statistical significance was set to a *P* value <0.05.

### 2.6. Real-Time Quantitative PCR

RNAs from dissected hippocampi (500 ng) were individually reverse transcribed into cDNAs overnight at 37°C using the Verso cDNA kit (ThermoFisher Scientific, Waltham, USA) according to the manufacturer's instructions. qPCR assays were performed in a Lightcycler 480 System (Roche), in the presence of 200 nM of each primer: (*c-Fos* 5′agggagctgacagatacactcc3′_forward and 5′tgcaacgcagacttctcatc3′_reverse; *Homer1* 5′gatggagctgaccagtaccc3′_forward and 5′tggtgtcaaaggagactgaaga3′_reverse; *Ifnar2* 5′ggacagcgttaggaagaagc3′_forward and 5′tggaagtaagtctctaaggacaaatg3′_reverse; *Egr2* 5′ctacccggtggaagacctc3′_forward and 5′aatgttgatcatgccatctcc3′_reverse; *Bdnf* 5′agtctccaggacagcaaagc3′_forward and 5′tgcaaccgaagtatgaaataacc3′_reverse; *Arc* 5′ggtgagctgaagccacaaat3′_forward and 5′ttcactggtatgaatcactgctg3′_reverse; *Sod1* 5′caggacctcattttaatcctcac3′_forward and 5′tgcccaggtctccaacat3′_reverse; *pPib* 5′ttcttcataaccacagtcaagacc3′_forward and 5′accttccgtaccacatccat3′_reverse for normalization), 100 nM of specific hydrolysis probe (designed with Universal Probe Library, Roche Applied Science) and 1X Lightcycler 480 Probes Master mix (Roche, France) and normalized using the Lightcycler 480 SW 1.5 software. Data were analyzed using an analysis of variance (ANOVA) with two main factors: genotype (Ts65Dn versus euploid) and treatment (*α*5IA versus vehicle), and Fisher's post hoc complementary analysis was carried out when required by the experimental design to assess complementary statistical effects. Pearson correlation coefficients between IEGs expression and behavioral data (mean distance travelled during the first three days trial of the MWM task) were calculated. All analyses were performed using Statistica v6 (StatSoft, Inc., Tulsa, Okla, USA) or GraphPad Prism (GraphPad Software, La Jolla, Calif, USA) softwares. Statistical significance was set to a *P* value <0.05.

## 3. Results

### 3.1. Gene Expression Profiles after Treatment with *α*5IA in Euploid and Ts65Dn Mice Hippocampi

**Figure 2:**
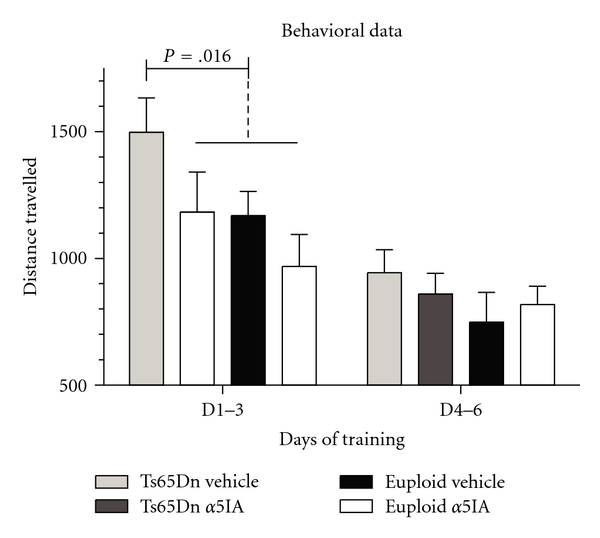
Effects of *α*5IA treatment in Ts65Dn mice trained in the MWM task. TS65Dn mice had impaired performance in the MWM task (comparison with other groups: *P* < .025). This deficit was rescued by treatment with *α*5IA.

We showed previously that Ts65Dn mice are impaired in the MWM task and that their learning proficiency can be restored following *α*5IA treatment [35]. In the present study, mice were trained in the MWM task using a similar protocol and treated daily with *α*5IA (5 mg/kg). Behavioral data were analyzed although the number of animals was small (5 or 6 per group) and precluded any robust statistical analysis (ANOVA). During the three first training sessions, vehicle-treated Ts65Dn mice travelled a longer distance to find the platform as compared to *α*5IA-treated Ts65Dn mice or euploid mice treated with vehicle or *α*5IA (*t*_16_ = 7.23; *P* = .016; Figure 2). Thus, as previously described in [35], we showed that behavioral deficit of the Ts54Dn mice in the MWM task was recovered following *α*5IA treatment. Twenty five minutes after completion of this long-term behavioral stimulation (12 days), mice were sacrificed and their hippocampi dissected. RNAs were extracted and amplified, labeled using *in vitro* transcription, and then hybridized on microarrays.

Among 41,000 genes present on the microarray, 13,024 were found to be expressed. Data were normalized and analyzed using ANOVA with two factors: genotype and treatment (Table 1). We found an effect of genotype (euploid versus Ts65Dn) on 848 genes representing 4.52% of whole genes expressed, an effect of treatment (vehicle versus *α*5IA 5 mg/kg) on 781 genes (4.17%) and a genotype-treatment interaction effect on 1,260 genes (6.73%). Principal component analysis (PCA) using all the genes expressed did not reveal any segregation of animal groups (data not shown), indicating that genotype and treatment did not globally affect expression profiles. Among the differentially expressed genes, we searched for enrichment of genes belonging to particular gene ontology (GO) categories. As shown in Table 2, 17 and 19 GO categories were significantly enriched among genes differentially expressed according to genotype and treatment, respectively. Eighteen of these GO categories were directly related to neurogenesis (Table 2 in bold).

We then analyzed the expression data focusing on genes of interest such as the expressed genes that are in three copies in Ts65Dn mice (3N genes, *n* = 56) and immediate early genes (IEGs, *n* = 16) which are involved in active memorization processes, and the product of which was shown to be increased by *α*5IA treatment in a previous study [35].

### 3.2. Gene Expression Changes of 3N Genes after *α*5IA Treatment

**Figure 3:**
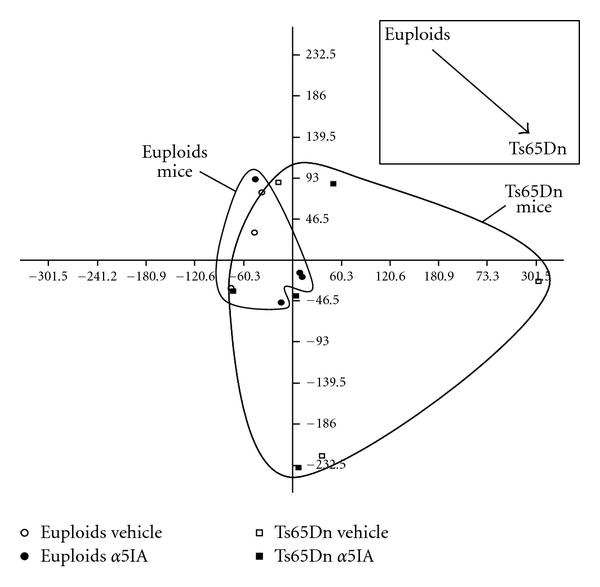
PCA on three-copy genes from Ts65Dn mice measured on microarrays. The first two principal components discriminated between euploid and Ts65Dn mice.

PCA on the 56 3N genes expressed in hippocampus showed a partial segregation between euploid and Ts65Dn mice (Figure 3). Expression levels of 3N genes were significantly different in Ts65Dn mice as compared to euploid mice (*t*-test *P* < .05) with a mean ratio of 1.13 and 1.10 between ts65Dn and euploid mice under vehicle or *α*5IA treatment, respectively, suggesting a global increase of expression of three-copy genes. ANOVA with two factors (genotype and treatment) specifically on 3N genes revealed an effect of genotype on 6 genes that represented 10.71% of the total number of expressed triplicate genes (56): *Gart, Ifnar-2, Kcnj6, Itsn1, Hlcs*, and *Sod1* (Table 1). The mean expression ratio Ts65Dn/Euploid for these 6 three-copy genes was found to be 1.22 (*t*_12_ = 3.49; *P* = .0045). In addition, we found that *α*5IA treatment impacted on the expression levels of 3 three-copy genes (*App, Kcnj6,* and *Sod1*). Interactions between genotype and treatment were significant for 5 genes (*Cbr1, Gabpa*, 4931408A02Rik, *Hmngn1,* and *Pcp4*). Among differentially expressed genes, there was no significant enrichment of 3N genes. However, a strong tendency to overrepresentation of three-copy genes modulated by the genotype factor in comparison to the overall distribution was observed (chi-square: *P* = .057; Table 1).

**Figure 4:**
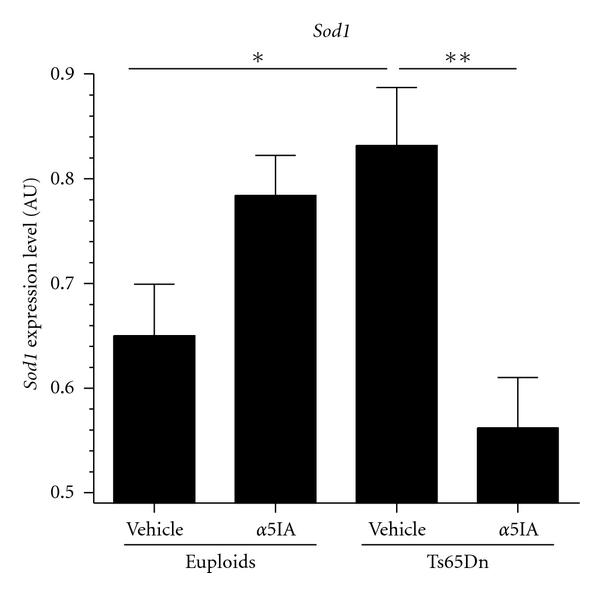
QPCR expression level of *Sod1* gene in euploid and Ts65Dn mice after chronic treatment with vehicle or *α*5IA. *P < .05, **P < .01, and two-way ANOVA with Fisher's post hoc comparisons.

Among these genes modulated by the genotype factor, our attention was particularly drawn to the *Sod1* gene whose role in DS was largely demonstrated [38, 39]. In order to confirm the microarray results, QPCR analysis was performed on RNAs from 13 mice (3-4 per group) used for microarray and on RNAs from onther 8 mice (2 per group). Two-way ANOVA analysis on *Sod1* gene showed a treatment by genotype interaction effect (*F*_1,17_ = 16.77; *P* < .00005; Figure 4). *Sod1* expression level of vehicle-treated Ts65Dn mice increased in comparison to vehicle-treated mice (*F*_1,17_ = 7.01; *P* < .05) with a mean ratio of 1.27 (Figure 4). Treatment reduced the level of *Sod1 *expression in *α*5IA-treated Ts65Dn mice (*F*_1,17_ = 14.43; *P* < .01). Thus, *Sod1* expression in Ts65Dn mice under *α*5IA treatment was similar to vehicle-treated euploid mice, suggesting that chronic treatment with *α*5IA allowed *Sod1* to return to a physiological level of expression in the hippocampus of Ts65Dn mice.

### 3.3. Gene Expression Changes of IEGs Genes after *α*5IA Treatment

**Figure 5:**
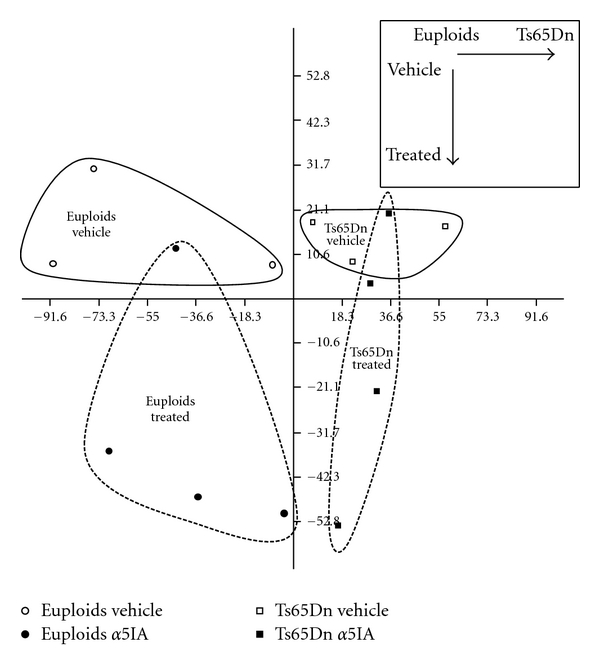
PCA on IEGs expression measured on microarrays. The first principal component fully discriminated euploid and Ts65Dn mice. The second principal component partially discriminated vehicle- and *α*5IA-treated mice.

PCA on the 16 IEGs expressed in the hippocampus showed a total separation between euploid and Ts65Dn mice but also a partial segregation as a function of treatment (Figure 5). Two-way ANOVA on these 16 IEGs revealed an effect of genotype (euploid versus Ts65Dn) on 5 genes (*Bdnf, Cox2, Homer1, RGS2,* and *Arc*), an effect of treatment (vehicle versus *α*5IA 5 mg/kg) on 3 genes (*c-Fos*, *Egr2,* and *Bdnf*), and a genotype by treatment interaction effect on one gene (*Bdnf*). Among the differentially expressed genes, there was a significant enrichment of IEGs modulated by genotype and treatment (chi-square: genotype *P* < .001, chi-square: treatment *P* < .05) supporting an effect of genotype and treatment on IEGs expression levels (Table 1). 

**Figure 6:**
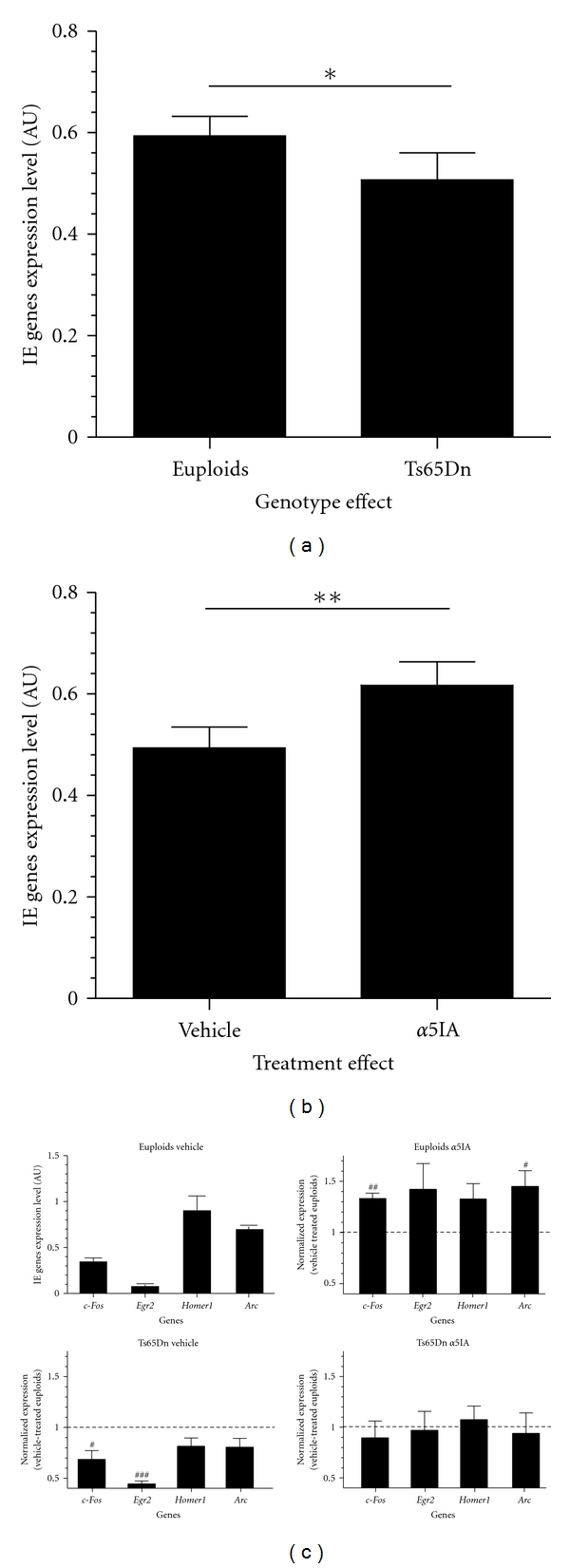
QPCR expression level of selected IEGs. (a) Mean cumulated expression levels of five selected IEGs in euploid and Ts65Dn mice; (b) effect of *α*5IA treatment on the mean level of expression of five selected IEGs; (c) expression of *c-Fos, Egr2, Bdnf, Homer-1,* and *Arc* in the four genotype and treatment experimental groups. *P < .05, two-way ANOVA with Fisher's post hoc comparisons; ^#^ *P* < .05, ^###^ *P* < .001, and one-sample *t*-test.

We selected 4 IEGs classically described as expressed during behavioral stimulation: *Arc*, *Homer1*, *c-Fos,* and *EGR2* for validation using QPCR analysis on 21 samples (5-6 per group), 8 of which were not used in the microarray experiments (Figure 6). Two-way ANOVA analysis with within-subjects design on IEGs expression showed genotype (*F*_1,16_ = 7.90; *P* < .05), treatment (*F*_1,16_ = 11.72; *P* < .01), and gene (*F*_4,64_ = 91.66; *P* < .001) effects. The expression level of IEGs was increased in euploid mice (treated or not with *α*5IA) as compared to Ts65Dn mice (Fisher's post hoc test: *P* = .012). The mean expression ratio Ts65Dn/euploid for these 4 genes was found to be 0.82. In contrast, *α*5IA-treated mice (euploid or Ts65Dn) showed higher levels of expression of IEGs relative to vehicle-treated mice (Fisher's post hoc test: *P* = .0034). The mean expression ratio *α*5IA/vehicle for these genes was 1.29.

**Figure 7:**
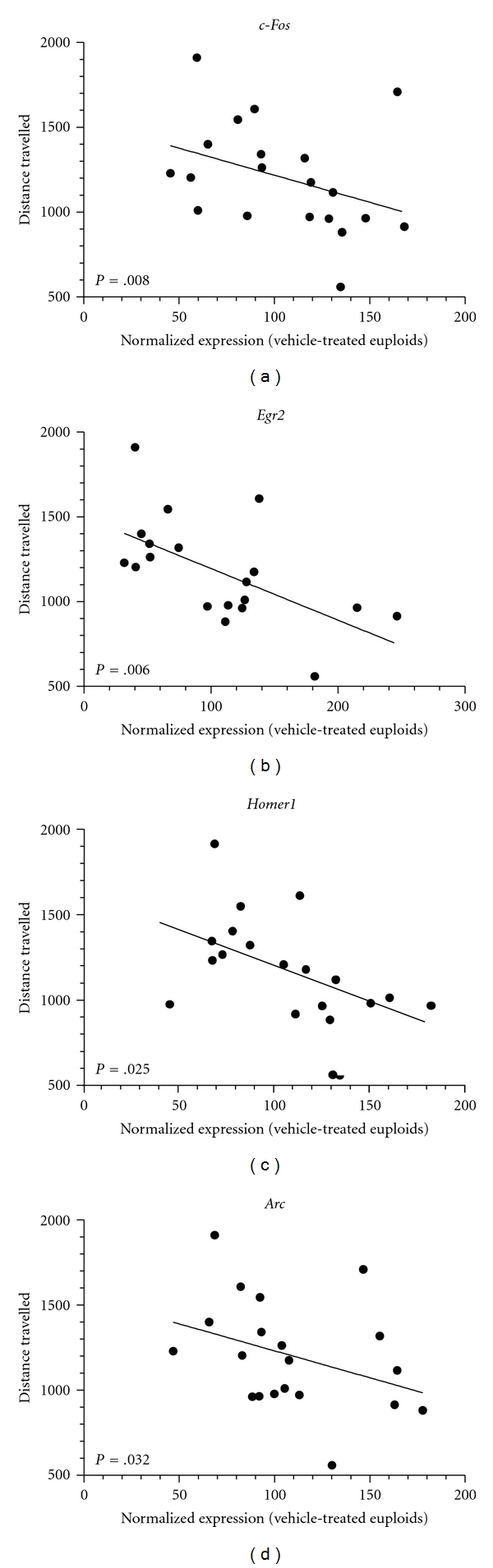
Correlation between IEGs expression levels and behavioral performances. Expression levels of IEGs were negatively correlated to the mean distance travelled during MWM testing (−0.645 < *r* < −0.494, Pearson correlation) underlining a tight relationship between learning proficiency and IEGs activation.

Individual IEGs expression levels were normalized against the vehicle-treated euploid mice value that corresponds to the physiological level of expression. In euploid mice, all IEGs increased after *α*5IA treatment. This increase was statistically significant for *c-Fos* and *Arc* genes (one sample *t*-test: *t*_4_ = 6.44, *P* = .003, and *t*_4_ = 2.89; *P* = .04, resp.). In Ts65Dn mice, the basal level of expression of IEGs was lower as compared to euploids. *C-Fos* and *Egr2* expression was drastically reduced (one sample *t*-test: *t*_3_ = 3.62, *P* = .036, and *t*_3_ = 20.38; *P* = .0003, resp.). Interestingly, IEGs expression profiles were normalized to euploid mice levels after *α*5IA treatment. In addition, we found inverse correlations between the expression levels of *Fos, Egr2, Homer1,* and *Arc* deduced from QPCR and the mean distance travelled during the first three training sessions of the MWM (−0.645 < *r* < −0.494; Figure 7).

## 4. Discussion

We have previously shown that treatment with *α*5IA alleviates learning and memory deficits of Ts65Dn mice [35] We also demonstrated that *α*5IA increased the expression of the IEG product Fos in specific brain regions involved in learning and memory following cognitive stimulation. Importantly, following *α*5IA administration, both genotypes were observed to display significant and comparable Fos induction. This potentiation of brain activity might therefore be the substratum of the general promnesiant effects of *α*5IA independently of the disease status. In order to gain more insight into mechanisms of the general promnesiant effects as well as the rescuing effects in Ts65Dn mice, we studied gene expression regulation networks in mice trained in the MWM task. During this continuous training episode, mice received daily injections of *α*5IA (5 mg/kg) for a total of 12 days. Gene expression was then measured using DNA microarrays from hippocampal RNA extracts obtained 30 min after the last training session.

### 4.1. Hippocampal Gene Expression Networks Regulated by *α*5IA in Control Euploid Mice

#### 4.1.1. Effects on HSA21 Genes

Microarray and QPCR analysis on the expression of genes from the region of mouse chromosome 16 which is triplicated in Ts65Dn mice and is orthologous to human chromosome 21 (HSA21) genes did not reveal any effect of chronic treatment with *α*5IA. These results suggest that genes from this triplicated region are not interfering with the activity of *α*5IA and hence do not modify the *α*5 subunit-containing GABA-A-benzodiazepine receptors or their signaling pathways.

#### 4.1.2. Effects on IEGs Expression during Memory Processes

Following memory stimulation, chronic treatment with *α*5IA enhanced IEG activation in euploid mice. It is likely that higher IEG transcripts following *α*5IA treatment will result in an increase of IEG protein products (e.g., Fos protein) as confirmed in our previous study [35], while without behavioral stimulation, *α*5IA did not increase the IEG product Fos (data not shown). These results suggest a state dependency (cognitive stimulation) of the effects of *α*5IA on IEG expression. IEG expression regulation after *α*5IA treatment following behavioral stimulation could be a contributing factor for both the general promnesiant activity of *α*5IA and its rescuing effect in Ts65Dn mice alongside signaling cascades that are critical for memory consolidation and cognition.

### 4.2. Hippocampal Gene Expression Networks Regulated by *α*5IA in Ts65Dn Mice

#### 4.2.1. Effects on HSA21 Genes

**Gene Expression Differences between Ts65Dn and Euploid Hippocampi**

Of the 108 HSA21 genes present on the microarray and expressed in the hippocampus, only 6 were differentially expressed between Ts65Dn and euploid mice: *Gart, Ifnar-2, Kcnj6, Itsn1, Hlcs *and,* Sod1*. To our knowledge, this is the first time that gene expression profiles have been established in the hippocampus of adult Ts65Dn mice. We focused our attention on the *Sod1 *gene which has been extensively studied in DS and confirmed our results using QPCR. LTP deficits observed in Ts65Dn [8] could be due to overexpression and thus increased activity of *Sod1* in the hippocampus since overexpression of *Sod1* gene in transgenic mice is sufficient to impair LTP [40]. Increased level of *Sod1* has also been shown to enhance the sensitivity to degeneration and apoptosis leading to a reduction of hippocampal neuronal progenitors [41]. This could explain enrichment for numerous GO categories related to neurogenesis among the genes differentially expressed between Ts65Dn and euploids. Such effects on neurogenesis-related genes may contribute to the memory deficits observed in Ts65Dn mice [42] and also in humans with memory dysfunction [43]. Since *Sod1* and IEGs expressions are inversely modulated in Ts65Dn mice as compared to euploids before and after treatment, it could also be speculated that *Sod1* and IEGs are functionally regulated, IEGs inhibiting *Sod1* expression and conversely increased *Sod1* levels decreasing IEGs after behavioral stimulation.

**Chronic Treatment with *α*5IA Restores the Expression of *Sod1*in Ts65Dn Mice to Normal Physiological Levels**

Chronic treatment of Ts65Dn mice with *α*5IA normalized the level of expression of *Sod1 *in the hippocampus. Although the exact mechanisms responsible for this effect are unclear, we can postulate that chronic treatment with *α*5IA could alleviate cognitive deficits at least partly through the normalization of *Sod1* expression in the hippocampus. Since *Sod1* overexpression impairs hippocampal neurogenesis and long-term synaptic plasticity, *α*5IA could reverse these deleterious effects by decreasing the expression levels of *Sod1*. Stimulating or restoring neurogenesis might thus participate in the recovery of cognitive functions of Ts65Dn mice, as suggested also by enrichment of GO categories associated with proliferation and cell death among differentially expressed genes.

#### 4.2.2. Effects on IEGs Expression

**Reduction of IEG Activation Pattern in Ts65Dn Mice**

It is known that IEGs play a key role in learning and memory mechanisms which are deficient in Ts65Dn mice. Indeed, long-term memory requires activation of specific IEGs [44]. Neuronal IEGs mostly code for transcription factors, growth factors, cytoskeletal proteins, metabolic enzymes, or proteins involved in signal transduction [45]. Memorization of new information requires the establishment of a pattern of IEGs expression. It has been shown that age-related memory deficits in rats result in an overall decrease in the expression of IEG in the hippocampus, particularly *Homer-1*, *Arc*, and different members of EGR family [46]. After behavioral stimulation in the MWM task, we found, using microarray and QPCR, a significant reduction in the overall level of IEGs in Ts65Dn mice as compared to euploids. This reduction was observed in particular for *c-Fos* and *Egr2*. For *Homer-1*, and *Arc,* decreased expression in Ts65Dn mice was just below the level of significance. This could be due to differences in the kinetics of waves of expression of these particular IEGs [44].

**Chronic Treatment of Ts65Dn Mice with *α*5IA Restores Normal IEG Activation Pattern**

Ts65Dn mice treated chronically with *α*5IA showed normalized levels of activation of IEG following memory stimulation, particularly *c-Fos, Egr2, Homer-1* and *Arc* that could be related to the recovery of MWM performance deficits observed in the present study and demonstrated previously [35]. Normalization of the activation profile of IEG following behavioral stimulation could thus be responsible for the rescuing effects of *α*5IA observed in Ts65Dn mice. The close relationship between IEGs expression levels and cognitive performances was indeed suggested by the significant inverse correlations found between the expression levels of *Fos, Egr2, Homer1* and *Arc* and performances in the MWM task. In addition, *α*5IA treatment was shown to restore Ts65Dn mice performances in the MWM task and to normalize the expression levels of *c-Fos, Egr2, Homer-1*, and *Arc*.

### 4.3. General Promnesiant and Rescuing Effects of *α*5IA

The general *α*5IA promnesiant effect observed in euploid and Ts65Dn mice could be explained by the acute pharmacological action of the drug directly on *α*5 GABA-A-benzodiazepine receptors. Indeed, *α*5 inverse agonists decrease GABAergic transmission and promote the excitability of postsynaptic neurons in rodents [28–32]. The previously described increase of Fos protein immunoreactivity after short-term memory stimulation combined with *α*5IA acute treatment could thus be the consequence of immediate release of GABA inhibition [35]. Following repetitive cognitive stimulations as in the MWM task that involves hippocampus-dependent memory, we evidenced IEGs activation deficits in the hippocampus of Ts65Dn mice that were rescued after chronic *α*5IA treatment. However, we cannot exclude that a single injection of *α*5IA would have a similar effect on IEGs expression levels. It thus appears that the rescuing effects of *α*5IA on long-term memories are more likely the consequence of hippocampal IEGs expression normalization than the long term effect of repetitive GABAergic modulations. In addition, as mentioned above, it is likely that the normalization of *Sod1* overexpression by *α*5IA is also important to promote cognitive rescuing.

## 5. Conclusions

We have identified genomic changes related to chronic treatment with *α*5IA, an *α*5-selective GABA-A receptor inverse agonist. Under physiological conditions in which *α*5IA has been shown to be promnesiant, increase of IEGs activation has been observed and in particular of *c-Fos* and *Arc* genes. This increase of activation could allow a more efficient storage of information during memory process.

Under pathological conditions such as DS in which deficits in learning and memory have been described, we were able to demonstrate an effect of chronic treatment with *α*5IA at the level of expression of different genes in Ts65Dn mice. Indeed, chronic administration of *α*5IA restored a normal level of *Sod1 *expression which is involved in hippocampal neurogenesis and LTP. In addition, chronic treatment with *α*5IA normalized the pattern of IEGs activation that is deficient in Ts65Dn mice. These genomic changes observed after chronic treatment with *α*5IA could be responsible for the restoration of learning and memory functions in Ts65Dn mice.

## Figures and Tables

**Table 1:** Analysis of variance (ANOVA) of microarray data: genotype (Euploids versus Ts65Dn mice) and treatment (Vehicle versus *α*5IA-treated) were the two main factors. Three-copy genes and IEGs were analyzed separately.

		Analysis of variance (ANOVA) of microarray data		
	Total	Genotype-modulated genes	Treatment-modulated genes	Interaction-modulated genes
All genes	13024	848	781	1260
3N genes	56	6	3	5
	Genes	*GART*, *Ifnar-2*, *Kcnj6*, *Itsn1*, *Hlcs*, and *Sod1 *	*App*, *Kcnj6*, and *Sod1 *	*Cbr*1, *Gabpa*, 4931408A02Rik, *Hmngn1*, and *Pcp4 *
IEGs and *BDNF *	16	5(***)	3(*)	1
	Genes	*BDNF*, *Cox2*, *Homer1*, *GS2*, and *Arc *	*Fos*, *Egr2*, and *BDNF *	*BDNF*

**Table 2:** Analysis of GO categories among the genes differentially expressed in the hippocampus of Ts65Dn mice after *α*5IA and behavioral stimulation. In bold GO categories related to neurogenesis processes.

GO term	Description	*P* value
Enrichment analysis of GO biological processes associated with genotype		
GO:0051272	Positive regulation of cellular component movement	2.93*E* − 4
**GO:0007216**	**Metabotropic glutamate receptor signaling pathway**	2.96*E* − 4
GO:0032879	Regulation of localization	3.3*E* − 4
**GO:0016265**	**Death**	3.55*E* − 4
GO:0009798	Axis specification	3.58*E* − 4
GO:0006414	Translational elongation	3.77*E* − 4
**GO:0030335**	**Positive regulation of cell migration**	4.21*E* − 4
**GO:2000147**	**Positive regulation of cell motility**	4.21*E* − 4
**GO:0008219**	**Cell death**	4.49*E* − 4
GO:0065008	Regulation of biological quality	4.66*E* − 4
**GO:0012501**	**Programmed cell death**	5.3*E* − 4
**GO:0008624**	**Induction of apoptosis by extracellular signals**	5.44*E* − 4
GO:0040017	Positive regulation of locomotion	7.32*E* − 4
**GO:0008283**	**Cell proliferation**	7.77*E* − 4
GO:0000578	Embryonic axis specification	7.96*E* − 4
**GO:0006916**	**Antiapoptosis**	8.14*E* − 4
**GO:0007049**	**Cell cycle **	9.3*E* − 4

Enrichment analysis of GO biological processes associated with *α*5IA treatment		
GO:0046883	Regulation of hormone secretion	4.35*E* − 5
**GO:0030335**	**Positive regulation of cell migration**	4.89*E* − 5
**GO:2000147**	**Positive regulation of cell motility**	4.89*E* − 5
GO:0040017	Positive regulation of locomotion	9.19*E* − 5
GO:0051272	Positive regulation of cellular component movement	1.04*E* − 4
**GO:0048869**	**Cellular developmental process**	1.49*E* − 4
**GO:0007176**	**Regulation of epidermal growth factor receptor activity**	3.38*E* − 4
**GO:0040008**	**Regulation of growth**	5.16*E* − 4
GO:0051270	Regulation of cellular component movement	5.9*E* − 4
**GO:0000302**	**Response to reactive oxygen species**	5.98*E* − 4
**GO:0030334**	**Regulation of cell migration**	6.81*E* − 4
GO:0048519	Negative regulation of biological process	7.57*E* − 4
**GO:0009888**	**Tissue development**	8.46*E* − 4
GO:0006012	Galactose metabolic process	9.12*E* − 4
GO:0009896	Positive regulation of catabolic process	9.15*E* − 4
**GO:0030154**	**Cell differentiation**	9.24*E* − 4
**GO:2000145**	**Regulation of cell motility**	9.33*E* − 4
GO:0035413	Positive regulation of catenin import into nucleus	9.67*E* − 4
GO:0031331	Positive regulation of cellular catabolic process	9.98*E* − 4
